# Case Report: Photodynamic therapy meets Chinese herbal medicine: a promising approach for advanced hypopharyngeal carcinoma

**DOI:** 10.3389/fphar.2025.1602910

**Published:** 2025-12-03

**Authors:** Mingsheng Lyu, Huayang Wu, Weixia Yu, Hang Zou, Chengjun Ban, Lu Wang, Hongwu Wang, Linyang Wang

**Affiliations:** 1 Department of Respiratory, The Third Affiliated Hospital, Beijing University of Chinese Medicine, Beijing, China; 2 Respiratory Disease Center, Dongzhimen Hospital, Beijing University of Chinese Medicine, Beijing, China

**Keywords:** Chinese herbal medicine, external therapy, hypopharyngeal squamous cell carcinoma, palliative care, photodynamic therapy

## Abstract

Hypopharyngeal squamous cell carcinoma (HPSCC) continues to have the bleakest prognosis among head and neck cancers. Managing inoperable HPSCC, particularly in elderly patients, presents a complex therapeutic challenge. This case details an 84-year-old female with advanced recurrent HPSCC and a complex medical history was treated using photodynamic therapy (PDT) combined with Chinese herbal medicine (CHM). She was presented with a hemorrhagic mandibular mass. PDT was administered due to her inability to undergo surgery. Despite significant side effects such as erythema and swelling from PDT, these were effectively managed with the external application of the CHM formula Jiedu Zhitong Powder, which alleviated pain and inflammation. The treatment led to a decrease in tumor size, resolution of ulceration and hemorrhage, and improved hemoglobin levels, allowing the patient to be discharged. Regular follow-ups showed no recurrent bleeding, although she eventually succumbed to complications related to tumor progression 7 months post-treatment. This case highlights the potential benefits of integrating PDT with CHM in treating unresectable HPSCC. The successful management of PDT side effects with CHM could represent a promising avenue for enhancing patient tolerance and adherence to treatment in HPSCC cases.

## 1 Introduction

Hypopharyngeal squamous cell carcinoma (HPSCC) represents 3%–5% of all head and neck cancers. Notably, between 60%–85% of HPSCC cases are diagnosed at advanced stages (III-IV) (Tsai et al., 2020). Although traditional treatments such as surgical resection, radiation, and systemic therapy have enhanced patient survival rates, HPSCC continues to have the bleakest prognosis among head and neck cancers with a 5-year overall survival rate of only 30%–40% (Luo et al., 2022). This alarming statistic underscores the critical need for innovative and multidisciplinary treatment approaches.

Photodynamic therapy (PDT) is particularly advantageous for treating head and neck disorders, especially for the unresectable, localized tumors prevalent in various head and neck squamous cell carcinoma (Marchal et al., 2015). Despite its benefits, PDT is frequently associated with severe side effects such as erythema, pain, burns, edema, and itching during light exposure, tremendously diminishing patient satisfaction and limiting its wider application. In response to these challenges, Chinese herbal medicine (CHM) emerges as a favored complement in China due to its minimal side effects and multi-target therapeutic properties. This paper discusses a case involving an advanced recurrent HPSCC patient with a hemorrhagic left mandibular mass who experienced significant pain post-PDT. The external application of CHM substantially alleviated the local inflammation and pain. Although this focal intervention did not alter the cancer’s progression, it enabled the patient to live an additional 16 months without recurrent bleeding, highlighting the potential of integrating CHM with conventional therapies. This case is pioneering in demonstrating the combined use of PDT and CHM in treating HPSCC, suggesting a viable, integrative approach to managing this advanced recurrent HPSCC. This case adheres to the CARE Guidelines.

## 2 Case description

An 84-year-old female presented with a 6-month history of a hemorrhagic left mandibular mass and was admitted to the Department of Respiratory in Dongzhimen Hospital in Beijing on 12 October 2021. The patient was highly suspected as hypopharyngeal malignant tumor by another hospital in Beijing 26 months ago. PET/CT showed that the left wall of the pharyngeal was thickened with increased metabolic activity. Meanwhile, multiple highly metabolized lymph nodes in the neck were also found, some of which were significantly enlarged with a maximum diameter of 3.3 cm, suggesting a high possibility of malignant metastasis. No other tissues with abnormal metabolic activity were found in the brain, chest, and abdomen. Then the diagnosis of hypodifferentiated metastatic squamous cell carcinoma of the left cervical regional lymph node was confirmed by the fine-needle aspiration biopsy (FNAB), as well as HPSCC was clinically diagnosed. To deal with the mass, the patient had undergone 35 rounds of radiation therapy advised by otorhinolaryngology-head and neck surgery doctors, while specific radiation doses and fields were unclear. Despite initial improvements, the jaw mass gradually increased in size and developed ulceration and hemorrhage by April 2021. Before hospitalization, her hemoglobin dropped to the lowest level of 49 g/L, necessitating frequent interventions including dressing changes, norepinephrine plus Yunnan Baiyao local administration, and transfusion to manage bleeding and anemia, while hemorrhage recurred after withdrawal. Meanwhile, the patient had a complex medical history including a cerebral aneurysm, hypertension, chronic cardiac insufficiency, previous myocardial infarction, renal insufficiency, and type 2 diabetes, regularly administering clopidogrel and metoprolol for secondary prophylaxis. The patient was unable to recall detailed family chronic medical history due to her advanced age, but no family history associated with malignant tumors was reported.

**FIGURE 1:**
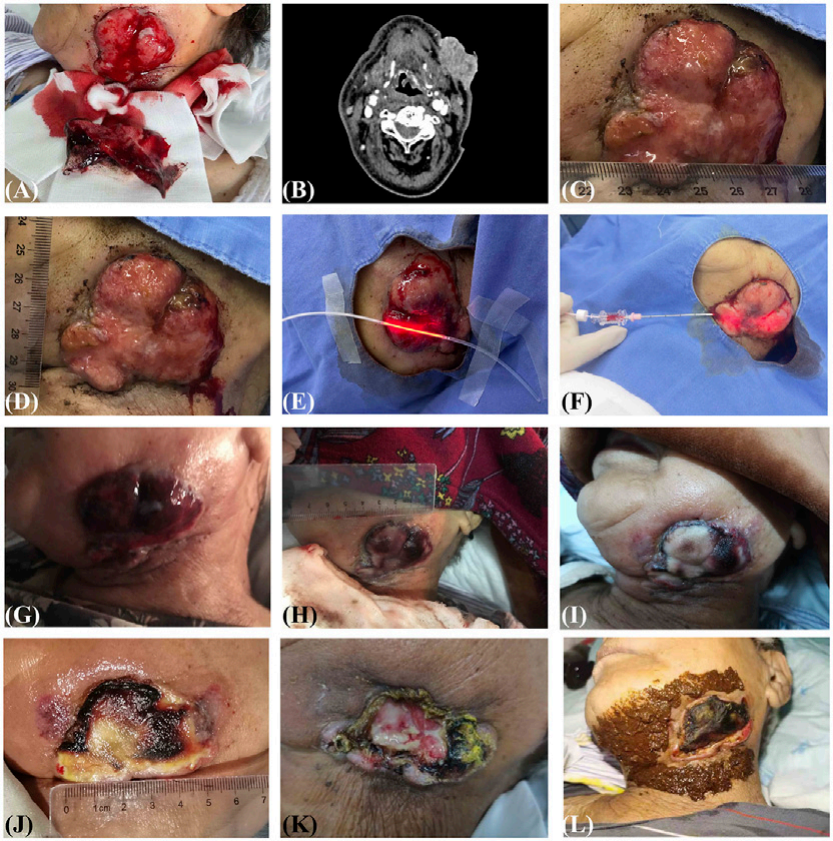
**(A)** A hemorrhagic red protruding mass on the left lower jaw (2021.09.28). **(B)** The enhanced neck CT scan indicated the left mandibular soft tissue tended to be malignant, with markedly uneven enhancement (2021.09.30). **(C–F)** The treatment process of PDT (2021.10.18). Measuring the size of pathological regions to decide specific surgical protocol **(C,D)**, including superficial PDT **(E)** and interstitial PDT **(F)**. **(G–K)** Postoperative changes of PDT. Acute local erythema, swelling, and pain were found around the tumor on the 2nd day **(G)**. As time went by, the ulceration and hemorrhage of the left mandibular mass were gradually relieved (H, the 3rd day; I, the 5th day; J, the 8th day; K, the 41st day). **(L)** External use of CHM around the affected area with the Guwei method according to TCM theory. CHM, Chinese herbal medicine; CT, computerized tomography; PDT, photodynamic therapy; TCM, traditional Chinses medicine.

Following admission, an uneven, hemorrhagic, ulcerative, protruding mass measuring approximately 5 cm × 6 cm × 4 cm in size was observed on her left lower jaw, but no pus or special odor was observed (Figures 1A–D). The enhanced neck computerized tomography (CT) scan indicated the left mandibular soft tissue tended to be malignant (Figure 1B), as well as multiple ground glass nodules in the lung, right axillary lymphadenectasis, and thickening of bilateral aryepiglottic fold were found by chest CT. Apart from moderate anemia (hemoglobin of 64 g/L) and renal insufficiency (serum creatinine of 133.7 mmol/L), no other abnormal laboratory findings were found. With her complicated comorbidities and poor general condition with an ECOG score of three points, she was deemed unfit for surgery under general anesthesia and also refused further invasive diagnostics aiming for other abnormal tissues. Meanwhile, the patient claimed to have suffered insufferable dry throat and pharyngalgia during the previous radiotherapy, leading to the refusal of further radiation therapy. Consequently, PDT under local anesthesia was chosen as the treatment method. We conducted the biopsy of the hemorrhagic left mandibular mass to identify its histopathology pre-procedure. It was observed that poorly differentiated cancer infiltration in the fibrous tissue, with morphological changes resembling squamous cell carcinoma. The immunohistochemistry staining revealed tumor cells positive for CK5/6, CK8/18, CK19, CK, WT1, and negative for CK14, CK20, TTF-1, NapsinA, TG. Hence, the patient was diagnosed with stage IVC advanced recurrent HPSCC (cT4aN2M1). PDT commenced with an intravenous administration of 2 mg/kg of hematoporphyrin (Hiporfin®) 48 h before laser application, then a semiconductor laser machine (PDT630-D2) was used to produce a 630 nm continuous wavelength light for activating the photosensitizer, with the power density of 100 mW/cm^2^ and energy density of 250 J/cm^2^. Given the large tumor volume and abundant blood supply, the staged irradiation protocol involved three sessions of multidirectional PDT using a 3 cm non-contact fiberoptic to perform both interstitial and superficial PDT, aiming to cover both the surface and deeper layers of the tumor (Figures 1E,F). The detailed plan of irradiation is listed in Table 1.

**TABLE 1:** Plan of PDT.

Date	Type of PDT	Number of irradiation areas	Irradiation time	Total irradiation dose
2021–10–18	Interstitial PDT	4	600 s	2400J
	Superficial PDT	5	600 s	3000J
2021–10–19	Interstitial PDT	3	600 s	1800J
	Superficial PDT	3	600 s	1800J
2021–10–20	Superficial PDT	3	600 s	1800J

On the second day after the initial PDT, the patient experienced significant local erythema, swelling, and pain around the tumor (Figure 1G). Given the patient’s red tongue and rapid slippery pulse, we prescribed the external CHM formula of *Jiedu Zhitong Powder*. Specific compositions of the CHM formula are listed in Table 2. To facilitate skin adhesion and active component release, the herbs were first mechanically ground with an electric grinder to obtain a fine, homogeneous powder of consistent particle size. The powdered formulation was then mixed with freshly extracted ginger juice as a solvent and excipient. Following filtration through a 200-mesh sieve to eliminate fibrous residues, the mixture was stirred to achieve a uniform, viscous, paste-like consistency, suitable for topical application without dripping. This paste was applied daily for 30 min around the affected area during the PDT treatment and continued for 2 weeks thereafter. All herbs of CHM formula had been certified as Good Agricultural Practice (GAP) by the National Medical Products Administration of China, which were obtained from the hospital’s pharmacy. In strict accordance with GAP guidelines, all crude herbs were traceable to their specific GAP-certified cultivation bases, which implement full-cycle standardized management from germplasm selection to harvesting and primary processing. This ensures correct botanical identity, consistent quality, and minimizes the risk of contaminants. The finished medicated powder was stored in airtight, light-protected containers at 4 °C to maintain stability. No other oral CHM or physical supportive therapy was applied during the treatment, and only symptomatic supportive treatments such as blood pressure control or glucose control were performed. This intervention was aimed at discharging heat, detoxifying, cooling the blood, and alleviating pain (Figure 1L). Following the application of the CHM formula, there was rapid relief of local inflammation and pain.

**TABLE 2:** Compositions of the *Jiedu Zhitong Powder*.

Formula	Compositions in Chinese name	Pharmacopoeial drug name	Daily dosage	Effects and mechanism
Jiedu Zhitong Powder	Huanglian	Coptis chinensis Franch. [Ranunculaceae]	20 g	1. Anti-inflammation effect 2. Analgesic effect 3. Antioxidant effect 4. Inhibition of vascular permeability 5. Reduce edema 6. Regeneration of skin collagen protein 7. Regulation of immune cells
	Huangbo	Phellodendron amurense Rupr. [Rutaceae]	20 g	
	Difuzi	Bassia scoparia (L.) Beck [Amaranthaceae]	20 g	
	Chishao	Paeonia lactiflora Pall. [Paeoniaceae]	20 g	
	Kushen	Sophora flavescens Aiton [Fabaceae]	20 g	
	Lianqiao	Forsythia suspensa (Thunb.) Vahl [Oleaceae]	20 g	
	Sangzhi	Morus alba L. [Moraceae]	10 g	
	Pugongying	Taraxacum sect. Taraxacum F.H.Wigg. [Asteraceae]	20 g	
	Lulutong	Liquidambar formosana Hance [Altingiaceae]	15 g	
	Zicao	Alternanthera sessilis (L.) DC. [Amaranthaceae]	10 g	
	Zihuadiding	Viola philippica Cav. [Violaceae]	15 g	
	Ruxiang	Boswellia sacra Flück. [Burseraceae]	10 g	
	Moyao	Commiphora myrrha (T.Nees) Engl. [Burseraceae]	10 g	

**FIGURE 2:**
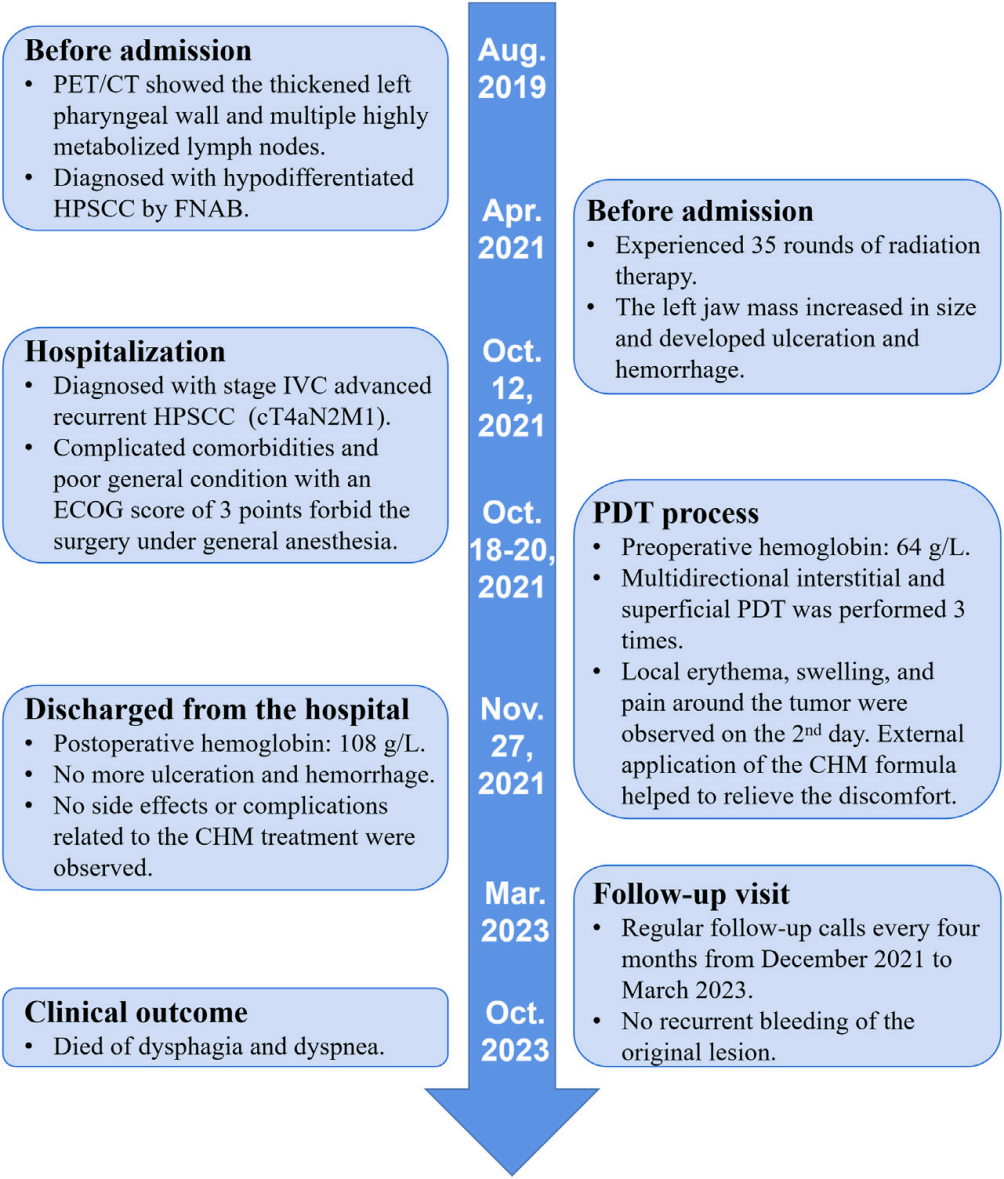
The timeline of this case. CHM, Chinese herbal medicine; CT, computerized tomography; FNAB, fine-needle aspiration biopsy; HPSCC, hypopharyngeal squamous cell carcinoma; PDT, photodynamic therapy.

PDT led to notable improvements in the patient’s condition. The ulceration and hemorrhage of the left mandibular mass were gradually relieved after three times PDT, with the overall volume of the tumor also decreased compared to before (Figures 1H–J). By the 41st day following the initial PDT session, the local lesion of the left jaw exhibited scabbing and hardening with evidence of granulation tissue regeneration, and no further ulceration or hemorrhage was observed (Figure 1K). The patient’s hemoglobin level improved to 108 g/L with no more transfusion during the hospitalization, as well as the activity of daily living scale score increased from 40 points upon admission to 60 points upon discharge, allowing for her discharge from the hospital, and she was orally administered the CHM formula in outpatient clinics. During the post-treatment phase, the patient did not experience any side effects or complications (e.g., contact dermatitis, systemic toxicity) related to the CHM treatment. Regular follow-up calls every 4 months from December 2021 to March 2023 confirmed no recurrent bleeding at the site of the original mandibular lesion. However, the patient finally died of dysphagia and dyspnea caused by the uncontrolled tumor compression 7 months later. The detailed timeline of interventions and outcomes is shown in Figure 2.

## 3 Discussion

Managing HPSCC, particularly in elderly patients, presents a complex therapeutic challenge due to the vital anatomical functions compromised by the tumor, including speaking, swallowing, and breathing. Based on the Chinese Society of Clinical Oncology (CSCO) guidelines for head and neck cancer (2021 edition), radiotherapy and chemotherapy (carboplatin or carboplatin) were regarded as first-line therapy. Whereas, the patient in this case claimed to have suffered insufferable dry throat and pharyngalgia during the previous radiotherapy, leading to refusal of further radiation therapy. We conducted an ECOG score on the patient, which was 3 points and deemed unsuitable for chemotherapy. So radiotherapy and chemotherapy were not an appropriate option. Immunotherapy was not considered due to its high cost of treatment. The application of PDT in this case accounted for its efficacy as a remarkable treatment modality for managing unresectable tumors, which meets previous studies about PDT in other inoperable cases of oropharyngeal and head and neck squamous cell carcinoma (Lambert et al., 2021).

PDT-mediated anti-tumor effect is multifactorial (Kazemi et al., 2024). On the one hand, with the activation by specific wavelength light, the photosensitizer molecule accumulates in the cancer cells and absorb the light to transform it from low-energy ground state (singlet state) to a higher-energy state (singlet-excited state), the excited photosensitizer transfers energy to oxygen to generate severe oxidative damages in forms of reactive oxygen species (Type I mechanism) and singlet oxygen (Type II mechanism), which leads to apoptosis and ultimately cell death. On the other hand, the irreversible destruction of the tumor vascular system is primarily responsible for an effective PDT of solid tumors. Besides, local inflammation induces blood flow stasis of capillary and platelet aggregation inside the tumor after lighting, which helps to trigger thrombosis and hematischesis of the tumor.

Despite its benefits, PDT is often accompanied by inevitable side effects such as pain, erythema, and edema, which lead to low adherence and significantly deter patients from continuing treatment. It was reported that cold air analgesia could partly control pain during PDT, while the low temperature of air might cause local vasoconstriction, reducing reactive oxygen singlets production and thus limiting clinical effectiveness (Fink et al., 2015). Other methods such as nerve blocks had potential adverse events including hematoma, dysesthesia, or paresis by direct nerve injury (Fink et al., 2015). In this paper, the combined administration of non-steroidal anti-inflammatory drugs and clopidogrel for elder patients increased the risk of gastrointestinal hemorrhage. Hence, it underscores the pressing necessity for more effective methods to manage side effects during PDT. In this report, we prescribed the CHM formula of *Jiedu Zhitong Powder* for external use, which was an experience formula of our center. According to previous studies, herbs in *Jiedu Zhitong Powder* have the function of anti-inflammation, analgesic properties, antioxidant, inhibition of vascular permeability, edema reduction, regeneration of skin collagen protein, and regulation of immune cells (Fan et al., 2024; Zhang and Shi, 2010; Wang et al., 2023; Guo et al., 2022). All these findings provide a supportive role in the patient’s treatment regimen by enhancing tolerance to PDT. However, this study was a single case report, and the effectiveness of combination of PDT and CHM required further medical evidence. We would conduct more prospective studies with a larger sample size to assess the impact of this intervention method on the long-term prognosis of HPSCC patients.

## Data Availability

The original contributions presented in the study are included in the article/supplementary material, further inquiries can be directed to the corresponding authors.
